# Congenital Proximal Radioulnar Synostosis in an Elite Athlete–Case Report

**DOI:** 10.3390/medicina59030531

**Published:** 2023-03-08

**Authors:** Ilja Chandoga, Róbert Petrovič, Ivan Varga, Boris Šteňo, Emὄke Šteňová

**Affiliations:** 1 Second Department of Orthopedy and Traumatology, Faculty of Medicine, Comenius University in Bratislava and University Hospital Bratislava, 85107 Bratislava, Slovakia; 2 Institute of Medical Biology, Genetics and Clinical Genetics, Faculty of Medicine, Comenius University in Bratislava and University Hospital Bratislava, 81108 Bratislava, Slovakia; 3 Institute of Histology and Embryology, Faculty of Medicine, Comenius University in Bratislava, 81372 Bratislava, Slovakia; 4 First Department of Internal Medicine, Faculty of Medicine, Comenius University in Bratislava and University Hospital Bratislava, 81369 Bratislava, Slovakia

**Keywords:** congenital radioulnar synostosis, elbow score, *SMAD6* gene, *NOG* gene, *GDP5* gene

## Abstract

*Background and Objectives*: Proximal radioulnar synostosis (PRUS) is the most frequent congenital forearm disorder, although the prevalence in the general population is rare with a few hundred cases reported. Pfeiffer, Poland, Holt–Oram, and other serious congenital syndromes contain this abnormality. Non-syndromic cases with isolated PRUS very often exhibit as SMAD6, NOG genes variants, or sex chromosome aneuploidy. A subgroup of patients with haematological abnormalities presents with HOXA11 or MECOM genes variants. *Case report:* We present a non-syndromic adult elite ice-hockey player with unilateral proximal radioulnar synostosis of the left forearm. In early childhood he was able to handle the hockey stick only as a right-handed player and the diagnosis was set later at the age of 8 years due to lack of supination. Cleary–Omer Type III PRUS was found on x-ray with radial head hypoplasia and mild osteophytic degenerative changes of humeroulnar joint. Since the condition had minimal impact on sports activities, surgical intervention was not considered. The player continued his ice-hockey career at the top level and joined a national team for top tournaments. Upper extremity function assessment with questionnaires and physical testing resulted in minimal impairment. The most compromised tool was the Failla score with 10 points from a total of 15. Genetic testing with Sanger sequencing revealed no significant pathogenic variant in *SMAD6, NOG,* and *GDP5* genes. No potentially pathogenic copy number variants were detected by array-based comparative genomic hybridization. *Conclusions:* In the reported case, the ability of an athlete to deal with an anatomic variant limiting the forearm supination is demonstrated. Nowadays, a comprehensive approach to rule out more complex musculoskeletal impairment and family burden is made possible by evolving genetics.

## 1. Introduction

Despite proximal radioulnar synostosis (PRUS) being a rare condition, it is one of the most frequent congenital elbow disorders [1]. Several publications cite approximately 350 globally described cases, but this figure has remained unchanged for more than two decades [1,2]. More relevant data are available in a recent systematic review of operated patients with this diagnosis, which found 374 operated forearms [3].

The first report of PRUS is dated to 1793 in Museum Anatomicus by Sandifort [4]. This skeletal abnormality is frequently present as a part of syndromes.

From an embryological point of view, the upper extremity forms from the upper limb bud during the period from 4th to 6–8th week of gestation. The elbow joint develops during day 34 and the forearm is formed between the 37th and 57th day of gestation from the common cartilaginous mesenchymal plate separated from distal to proximal direction and forming the radius and ulna. The disturbance of the longitudinal separation of the forearm into the radius and ulna leads to the creation of a fibrous junction between these two bones [5]. The ossification happens between 1 and 4 years old [6]. 

The involvement of PRUS might be found in patients with Pfeiffer syndrome or Poland syndrome [7,8]. In addition to the radial longitudinal deficiency (RLD), the PRUS was found in 15% patients with Holt–Oram syndrome where the Cleary–Omer Type II with reduced radial head appeared in all cases [9]. This segmentation defect is present as a feature of Klinefelter’s syndrome (47 XXY) and other sex chromosome abnormalities as well. Non-syndromic PRUS are autosomal dominant with variable penetrance and an inclination to be bilateral in over 60% [10,11,12]. It is often connected with another conditions such as hip dysplasia or other musculoskeletal anomalies [5]. 

In recent years there has been a high demand and recommendation to identify the genetic cause of PRUS. In patients with isolated PRUS, most commonly *SMAD6*, *NOG* variants, or sex chromosome aneuploidy are often reported present, while PRUS in patients with hematological abnormalities is more often joined with *HOXA11* or *MECOM* genes variants [13]. NOG and SMAD6 proteins act as inhibitors of the bone morphogenic protein (BMP). BMP is known as a product of the ventral ectoderm, being important in differentiating the ventro-dorsal axis. The NOG protein is an extracellular and SMAD6 an intracellular inhibitor of BMP [14]. The nogging gene (*NOG*) is associated with NOG-related symphalangism spectrum disorder (NOG-SSD), enclosing proximal symphalangism, synostoses, stapes ankylosis with broad thumbs (SABTT), tarsal-carpal coalition syndrome, and brachydactyly [15,16]. The *SMAD6* gene mutations are supposed to play a role in non-syndromic craniosynostosis as well [17,18].

There are two classifications that are commonly used for this condition. The Wilkie classification distinguishes the Type I with proximal location of the synostosis and complete fusion of imperfectly formed radius to the ulna for a distance of several centimeters. The fusion in Type II is placed more distally and the radial head is often dislocated [19]. The Cleary–Omer classification describes four types of PRUS. Type I is a fibrous synostosis between the proximal ulna and radius, while in Type II there is an osseous synostosis, both with normal position of the radial head. Posterior dislocation of the hypoplastic radial head is present in Type III osseous synostosis while in Type IV, an anterior dislocation of a mushroom shaped radial head along with fibrous pseudo-synostosis is present [20].

Apart from congenital aetiology, the second possible cause of the synostosis between the ulna and radius is a fracture or surgery of the forearm. In this case, the cause is damage to the interosseous membrane resulting in the formation of a bony bridge. Overall, these cases are rare, and the X-ray findings are different. 

In this case, a rare case report of PRUS in an adult elite-level athlete with challenging demands of the upper extremity is described. The aim is to highlight the possibility of reaching the top sports level even with a congenital anomaly of the loaded limb. To our knowledge, there has not been a published case of a professional elite athlete with congenital radioulnar synostosis requiring affected upper limb activity.

## 2. Case Report

A 32-years old, a professional elite ice-hockey player with an unilateral proximal radioulnar synostosis of the left forearm is presented (non-dominant extremity). 

The patient remembers the inability to handle his first left-sided hockey stick in the correct position with the lower left hand in supination (left hand down). Playing was only possible with holding of the right-sided hockey stick (as a right-handed player with supinated right hand down on the stick). Despite this inconformity, the diagnosis was set later, at the age of 8 years.

The patient remembers a particular situation where he was not able to rotate his hand with the palm facing up and have a handful of small screws placed into his palm from another person. No family history of PRUS or other musculoskeletal impairments were known. In our patient, the post-traumatic radioulnar synostosis can be clearly ruled out due to a negative medical history of a fracture or surgery and typical X-ray findings for congenital aetiology. 

The surgical treatment was not indicated due to the absence of limitation in sport activities and the risk of interruption of the sport practice and surgical complications. The player continued his ice-hockey career at the top level and joined a national team as an adolescent. During his career, he participated in several world championships and Olympic games tournaments, not reporting significant difficulties with stick handling. 

### 2.1. Clinical Examination, Scoring, and Functional Testing 

**Figure 1:**
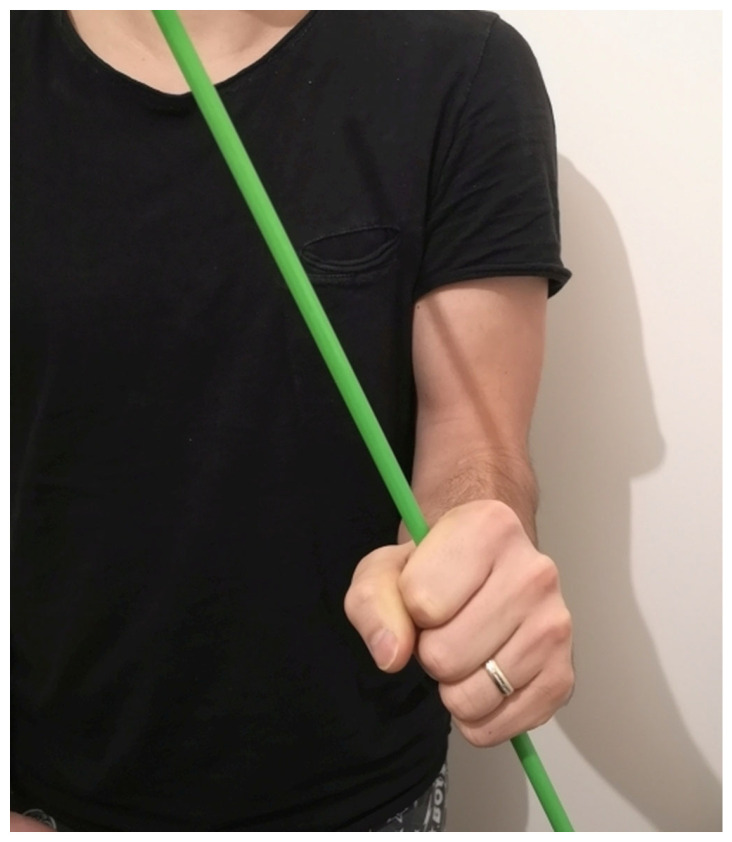
Forearm rotational range of motion where no real supination is possible. When trying to supinate, the result was 20° of pronation (minus 20° of supination).

**Figure 2:**
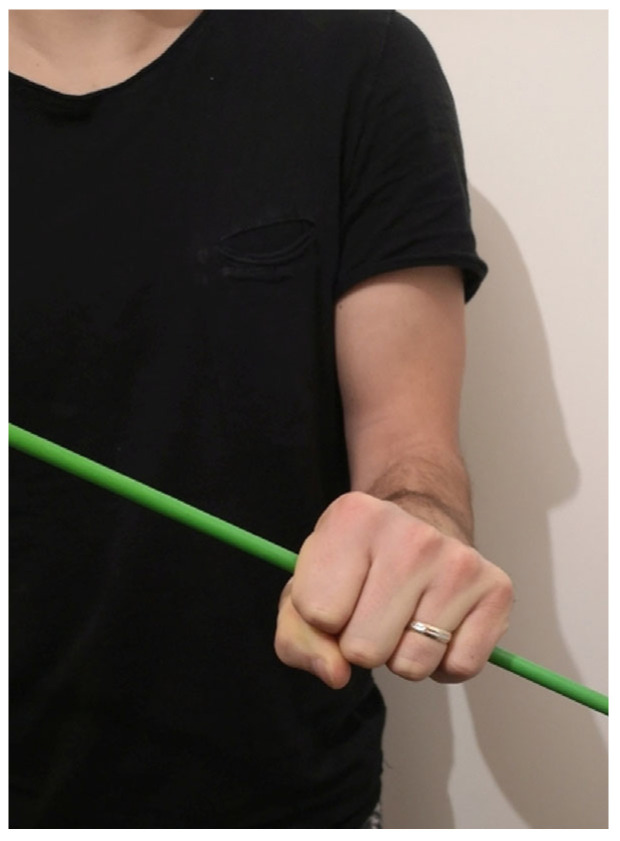
Forearm rotational range of motion with maximal pronation of 60°.

The objective clinical examination revealed no restriction of the elbow joint flexion and extension. The pronation range of motion was evaluated from the neutral position measured in 90° of flexion in the elbow. There was no real supination and the radioulnar rotational motion was from 20° to 60° of pronation (Figure 1 and Figure 2). 

After examination, questionnaires were performed to evaluate the function of the elbow joint and upper extremity.

The Mayo elbow score validation [21] of range of motion (ROM) and basic activities around the head resulted in normal left elbow joint function, achieving 100/100 points. 

The disabilities of the arm, shoulder, and hand (DASH) questionnaire revealed good function with 4.2 points from 100 (0 points means no disability). Some limitations that were derived from limited supination were present although did not impact the overall result [22]. 

The Broberg and Morrey rating system [23], measuring the arc of pronation and supination, evaluated a good result of 95 from a maximum of 100 points.

The Failla score [24], exploring handling and activities around the head, torso, and legs using the affected limb resulted in good result of 10 points from 15.

Physical performance testing was done by the closed kinetic chain upper extremity stability test (CKCUEST) [25]. The test is performed in push-up position with the hands located on separate pieces of tape at a distance 36 inches. The number of cross-touches of the hand to contralateral tape was 22 within 15 s.

### 2.2. Radiographic Evaluation

**Figure 3:**
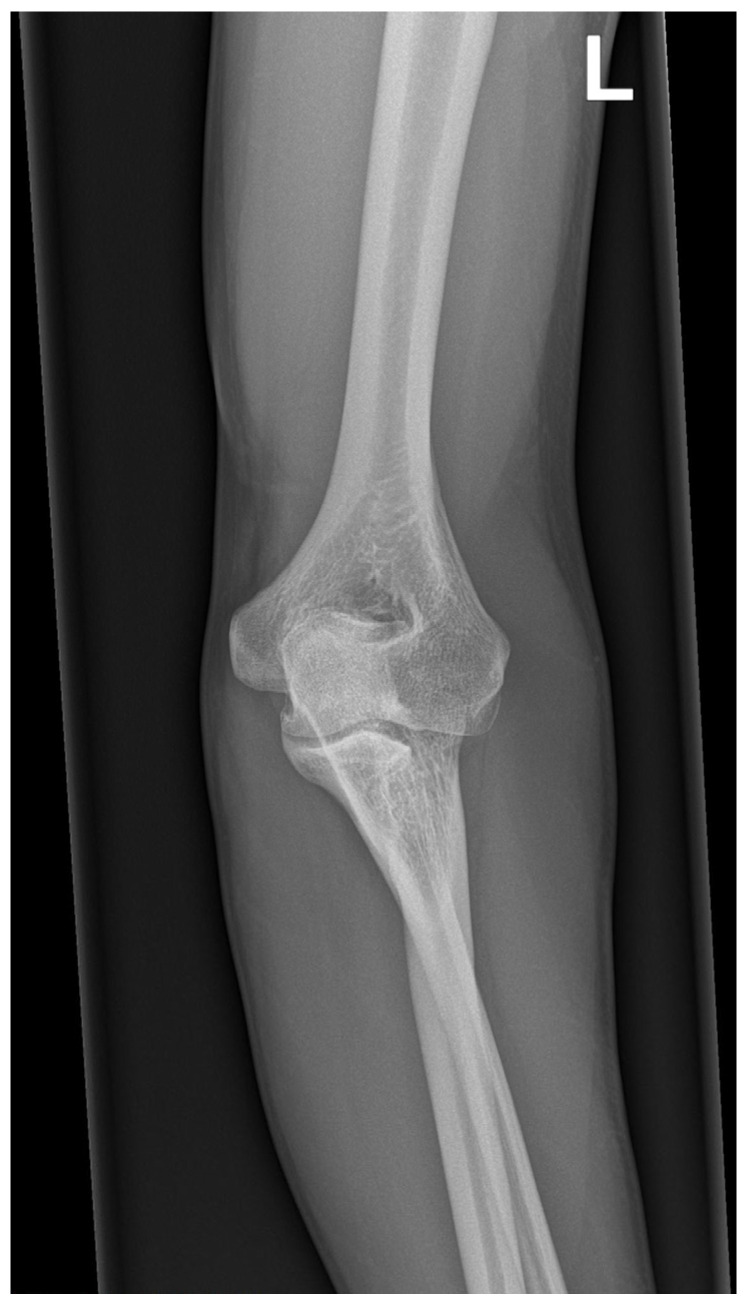
Radiographic image of the elbow joint in antero-posterior view showing Cleary–Omer Type III PRUS.

**Figure 4:**
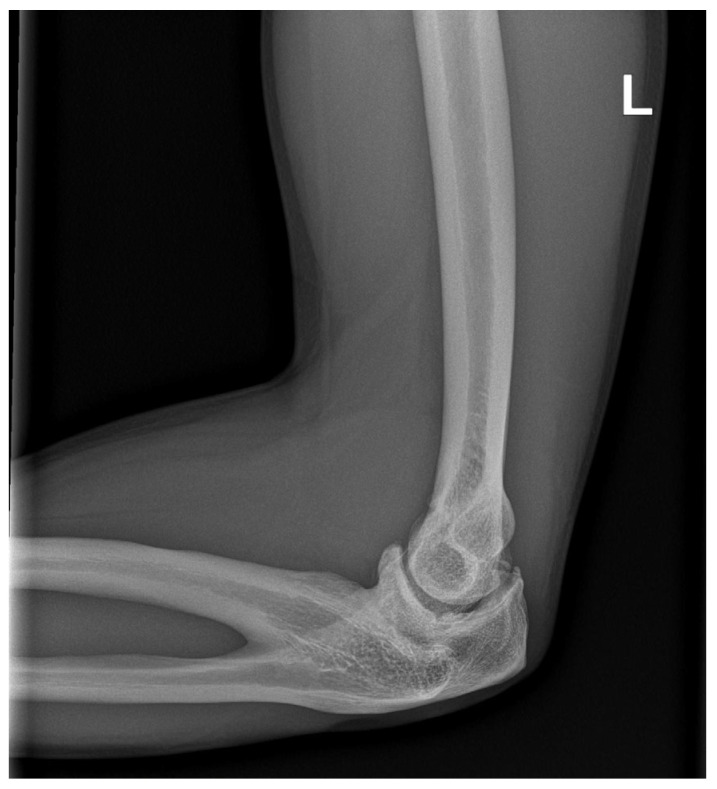
Radiographic image of the elbow joint in lateral view. Markable osteophytic changes of olecranon and coronoid process are present.

**Figure 5:**
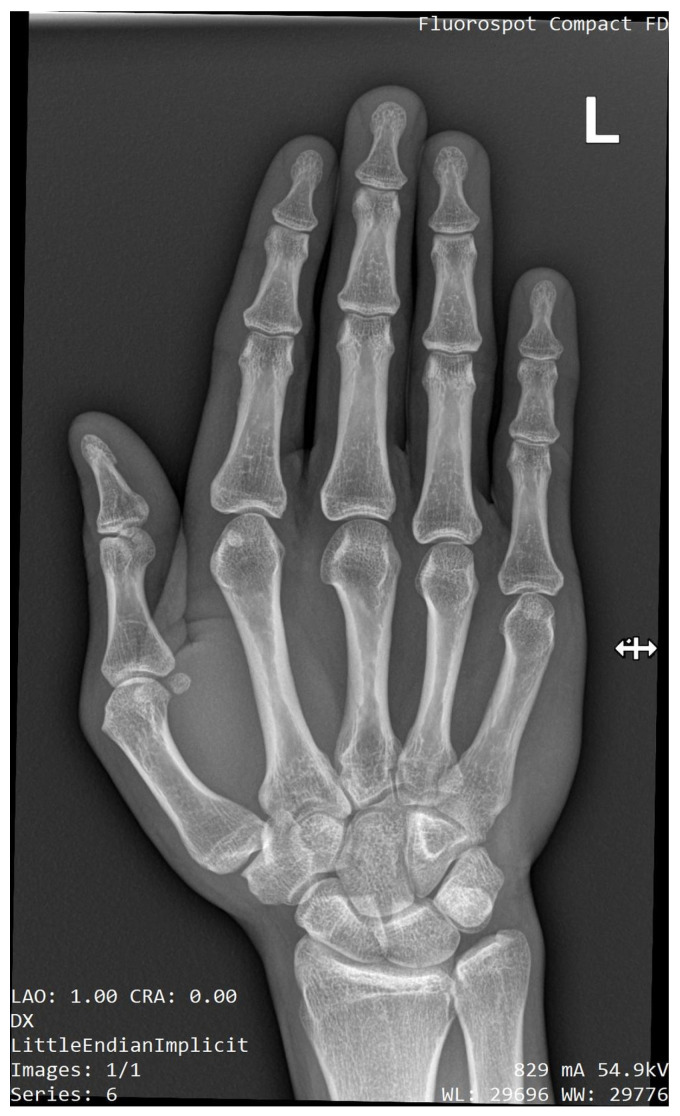
Radiographic image of the wrist joint in antero-posterior view showing hypoplasia of the ulnar styloid process.

X-ray imaging of the shoulder, elbow, forearm, and wrist was performed at the age of 32 years. A Cleary–Omer Type III PRUS was found on the antero-posterior and lateral X-ray with hypoplastic radial head. Osteophytic changes on coronoid and olecranon processes were present, indicating possible early degenerative changes (Figure 3 and Figure 4). The wrist antero-posterior X-ray revealed not clinically relevant hypoplastic ulnar styloid process (Figure 5). No apparent degenerative changes on the shoulder and wrist joint were found. 

### 2.3. Genetic Testing

Genomic DNA was extracted from peripheral blood by using a QIAamp DNA Blood isolation kit (Cat# 51104, Qiagene, Hilden, Germany) in accordance with the manufacturer’s protocol. Sanger sequencing was performed for the exons and intron–exon boundaries (+20 and −20 bp areas were included) of these genes: *SMAD6* (NM_005585), *GDF5* (NM_000557), and *NOG* (NM_005450). Polymerase chain reaction (PCR) amplification was performed using genomic DNA as a template and HOT FIREPol^®^ DNA Polymerase (Cat# 01-02-00500; SolisBiodyne, Ltd., Tartu, Estonia). The PCR conditions and primer sequences were described by Yang et al. [26]. Sanger sequencing was conducted using a BigDye^®^ Terminator v3.1 cycle sequencing kit (Applied Biosystems, Thermo Fisher Scientific, Inc., Waltham, MA, USA) in accordance with the manufacturer’s protocol. The amplified sequencing products were purified with 70% ethanol and then run on a SeqStudio Flex series genetic analyzer (Applied Biosystems, Thermo Fisher Scientific, Inc.). 

To search for copy number variants (CNVs), array-based comparative genomic hybridization (aCGH) was applied and performed according to the manufacturer’s instructions (SurePrint G3 Human CGH Microarray 8 × 60 K, Agilent Technologies, Santa Clara, CA, USA). Array images were acquired on the microarray scanner (Agilent SureScan Microarray Scanner, Agilent Technologies, Santa Clara, CA, USA). Data analysis was performed using the CytoGenomics software (v.5.1.2.1) (Agilent Technologies, Santa Clara, CA, USA). 

The obtained CNVs and sequencing variants were compared and classified with publicly available databases (Decipher, ClinVar, Database of Genomic Variants) in concordance with the American College of Medical Genetics and Genomics (ACMG) guidelines. As a reference the human genome assembly, 19 [hg19] was used.

No pathogenic variants in *SMAD6*, *GDF5,* and *NOG* genes were detected. We identified only one heterozygous variant in *SMAD6* gene that was classified as benign (c.120C>T, p.40Gly=, rs149612008). Also, no potentially pathogenic CNV was detected by aCGH (only some recurrent benign copy number variants were identified).

## 3. Discussion

Congenital proximal radioulnar synostosis is an inborn fibrous or bony bridge between the radius and the ulna, caused by impaired intrauterine longitudinal segmentation at the proximal part of the forearm [5]. The shoulder and wrist joint are able to compensate for the impaired supination in many cases [4]. 

The sequencing of genes underlying the non-syndromic PRUS was performed in our study. No pathogenic variant in *SMAD6, NOG,* and *GDF5* genes were detected and the aCGH analysis detected no genomic abnormalities. In our case, we investigated the most frequent genes that have been described to be associated with non-syndromic PRUS. Yang et al. performed a study of 140 patients and 11 families with non-syndromic radioulnar synostosis (ns-PRUS). Sex chromosome abnormalities were found in 10% from 140 sporadic patients. Rare variants of *SMAD6* were discovered in 19% (24/125) sporadic ns-PRUS cases and in 30% families (3/10) with ns-RUS [26]. Only one patient with RUS had *NOG* pathogenic variants. Another study confirmed the presence of variants in the *SMAD6* genes in 42.1% of cases from a cohort of 27 PRUS pedigrees and in 15.5% from a group of 251 PRUS sporadic patients and their family members [27]. 

Pathological variants of genes such as *MECOM* and *HOXA11* are present in patients with inherited hematological defects accompanied by platelet abnormalities, ranging from amegakaryocytic thrombocytopenia to bone marrow failure and myelodysplastic syndrome [28,29]. Both genes are involved in limb development and hematopoiesis. Walne et al. identified coding variants in *MECOM* in seven cases from five families with RUS and haematological abnormalities, two with autosomal dominant and three with de novo inheritance patterns [30]. Recently, Shen et al. reported MECOM-related PRUS without hematological aberration with unique variants in eight families [13]. However, these genes were not suspected in our case without a history of hematological abnormality. We cannot exclude the involvement of other genes in the aetiology of congenital RUS, which are not yet known and may be revealed through advanced methods such as wide genome sequencing. Diagnosis of a wider range of genes was beyond our capabilities. Patients with PRUS are not able to handle their daily activities that require supination. The abduction in the shoulder joint compensates for the lack of pronation and missing forearm supination is achieved with shoulder adduction [31]. Despite the ability to compensate for the lack of pronation and supination with the shoulder and radiocarpal joint, this mechanism fails in cases of severe hyper-pronation. The described forearm deformity affects several daily activities of patients. Patients are unable to accept coins and small objects in an open palm, comb their hair, or properly hold plates and cups [32]. Chopstick use may be limited in Asian countries. Patients report difficulty in manipulating smartphones, but typing on a PC keyboard is usually not a problem. Surgical intervention is currently indicated in preschool age, mainly for patients with severe hyper-pronation or bilateral PRUS. There are few published cases of similar presentation in adolescence or later in life, in which patients were aware of their limitation from childhood, but it was usually unilateral and well-compensated. During the athlete’s life, he was able to substitute the limitation of a non-dominant left hand with the right hand. In two described cases with unilateral PRUS, young women sought medical attention for elbow or wrist pain that was exacerbated by the need for increased care of small children [5,33]. Fortunately, excessive sports activities have not led to pain in our athlete. Only in exceptional cases, surgical intervention is described in later life [34].

In general, the surgical treatment is usually necessary in patients with fixed pronation over 60° [35]. An individualized approach is beneficial in patients with pronation between 15° and 60°. 

There are two possible types of surgery. To restore the movement in pronation and supination, the resection of synostosis with an effort to prevent the recurrence of the fusion is indicated. The second option is to change the hyper-pronation pattern to a more practical position with osteotomy. De-rotational surgeries, rather than motion-preserving surgeries, are performed in more than 90% of cases. In the recent meta-analysis containing 374 forearms, the surgical correction of supino-pronation was from a mean preoperative 64.8° pronation to a mean postoperative pronation of 2.8°. Reported complications after the surgery were radial nerve palsy, synostosis, compartment syndrome, radial head dislocation, radial shortening, delayed union/non-union, and infection. The only correlation that was observed between major complications and other relevant factors was the age of the surgery over 7 years of age [3]. A cohort of 12 patients from 4 to 6 years of age achieved satisfactory results with correction of pronation from 60°–85° to 20°–30°. Percutaneous fixation with K-wires was performed for osteotomy stabilization and no complications were recorded [36]. Similarly, the fixation may be achieved with plate fixation of both the radius and ulna as well. Hamiti et al. published 12 forearms in 10 patients (aged 3–9 years) achieving improvement of pronation from 35°–70° to 20°–25° with no complications [37]. To avoid the postoperative re-ankylosis after surgery the subcutaneous fat or free vascularized fascia lata graft may be used [38]. Considering the above results and complications of surgical treatment, it is evident that the athlete has so far been reluctant to undergo an operation for fear of negative consequences to his developing athletic career. In our case, the surgery is generally not recommended because the fixed pronation is not over 60°, the deformity is not bilateral or on dominant extremity [20].

When he started playing hockey as a child, he was unable to hold the hockey stick with his left hand down because he was unable to perform the supination movement; he easily adapted to playing with his right hand down. However, even this technical problem did not lead to a diagnosis being discovered.

In his further career, he did not notice any serious handicap when playing with his affected left hand up on the hockey stick. He was aware of the limitation in controlling the puck with the “toe-drag” technique, which requires supination of the upper forearm. However, he did not have any problems with shooting and passing. The situation would be completely different in the case of bilateral deformity, where he would not be able to perform the lower hand grip on the hockey stick. The player was unable to perform exercises with the underhand grip during conditioning practice on the pull-bar and dumbbells, not utterly necessary for an ice-hockey player.

A very important part of the case is that despite the daily performance load on the upper limb and compensating for the supination deficit, there was no development of degenerative or traumatic changes in his shoulder or wrist joint. Holding the hockey stick with the upper hand in palmar flexion of the wrist and pronation can lead to overloading the triangular fibrocartilage complex and its injuries [39]. In the case of fixed pronation and inability to change the wrist position to supination, we can assume increased stress on these structures. The player has not experienced any wrist pain so far.

Similarly, in the case of frequent shoulder injuries in contact sports (dislocations, rotator cuff injuries), the patient’s ability to compensate the forearm supination in the shoulder would be limited. Fortunately, the athlete has not had any left shoulder injuries so far.

The overuse of the underdeveloped elbow could potentially lead to early degeneration. X-rays revealed early signs of joint degeneration, but the player in our case has not reported any elbow pain yet. Additionally, during years of high-level activity, there have been no reports of elbow instability, which might be present with abnormal development of the radial head. In the presented case of clinically and radiologically apparent congenital skeletal abnormality, we sought to objectify and evaluate the degree of functional impairment. In addition, the case deals with an elite athlete whose needs in professional sport may be affected by even mild joint and movement limitations. Otherwise, with good results the athletes are able defend their functional performance despite an anatomical handicap.

DASH and Mayo elbow questionnaires are widely performed for upper extremity needs. As the PRUS is predominantly impeding the forearm supination, these scores may not be precisely sensitive for PRUS purposes, as we have also observed. The patient had unaffected extension and flexion in the elbow. The Failla score would be a more suitable tool to test the forearm rotation. Pei and Han operated on 36 forearms in 31 children where the mean initial pronation deformity of the PRUS was 62.92° ± 7.11° (range, 55° to 80°). Evaluating the Failla score, the excellent or good results increased from 5.6% preoperatively to 100% postoperatively. The mean Failla score increased significantly from 8.42 ± 1.42 (range, 5 to 10) points preoperatively to 14.89 ± 0.46 (range, 13 to 15) [24]. Our patient gained 10 points in the Failla score, corresponding to the boundary between a good and fair result. This result, together with the pronation between 20° and 60°, could impact the patient´s everyday life or other sporting activities. However, in the case of a right-sided ice hockey player (right hand down), this position of the left upper limb in pronation was satisfactory for him. Conversely, a correction of his left forearm toward the supination would likely make stick-handling difficult for him.

The CKCUEST may be performed as a screening test or a return-to-play tool in sportsmen and athletic population in painful conditions or after upper extremity injury and surgery. A number of repetitive touches less than 21 per 15 s may be predictive to sustain an injury during the season [25]. This examination confirmed good physical fitness of the affected upper limb even under body weight loading. In professional athletes, this would be crucial to defend their functional performance despite an anatomical handicap.

## 4. Conclusions

In our clinical case report of proximal radioulnar synostosis, the Failla score evaluated a markable functional deficit despite which the player is able to successfully perform a high-level professional career. Measuring the simple CKCUES test may be helpful to evaluate the athletes for competition when dealing with upper extremity musculoskeletal impairment. We found no mention in the literature of another described case of PRUS in an elite professional athlete and we intended to demonstrate his ability to deal with the anatomic variant. Despite the well tolerated functional limitation, no concomitant abnormalities that were detected and the absence of pathological variants in the most common genes, it is appropriate to point out the possibilities of a comprehensive approach, made possible by dynamically evolving genetics, to rule out more complex musculoskeletal impairment and family burden.

## Data Availability

The data that were presented in this study are available on request from the corresponding author. The data are not publicly available due to privacy restrictions.
